# Endothelial cell dysfunction: the culprit for cardiac denervation in aging?

**DOI:** 10.20517/jca.2023.36

**Published:** 2023-10-14

**Authors:** Oliver M. Moore, Joshua A. Keefe, Xander H. T. Wehrens

**Affiliations:** 1 Cardiovascular Research Institute, Baylor College of Medicine, Houston, TX 77030, USA.; 2 Department of Integrative Physiology, Baylor College of Medicine, Houston, TX 77030, USA.; 3 Department of Medicine (Cardiology), Baylor College of Medicine, Houston, TX 77030, USA.; 4 Department of Neuroscience, Baylor College of Medicine, Houston, TX 77030, USA.; 5 Department of Pediatrics (Cardiology), Baylor College of Medicine, Houston, TX 77030, USA.; 6 Center for Space Medicine, Baylor College of Medicine, Houston, TX 77030, USA.

**Keywords:** Arrhythmias, cardiac aging, denervation, endothelium, senescence

During cardiac development, nerves grow in close anatomic proximity to blood vessels due to their need for oxygen and nutrients. Vice versa, blood vessels require closeness to nerves for tight control of vasodilation and constriction. However, their interdependence also means that malfunction of one cell type may result in dysregulation of the other. Two important sources of cardiac innervation are the parasympathetic and sympathetic nervous systems, and under- or hyper-activity of either system can lead to heart failure or arrhythmias^[1]^. One common condition associated with autonomic nervous system deterioration and a predilection for cardiac arrhythmias is aging^[2]^. In a recent article published in *Science*, Wagner *et al.* demonstrated that aging-dependent vascular endothelial cell dysfunction reduces the density of neuronal axons in the heart, which in turn increases the risk of arrhythmias^[3]^.

For these groundbreaking studies, Wagner *et al.* utilized 18-20-month-old male and female wild-type (WT) mice as the primary model of aging^[3]^. Compared to young (3-month-old) mice, old mice exhibited ventricular diastolic dysfunction. Immunohistochemical staining of heart cross-sections revealed that all three major types of nerve fibers - parasympathetic, sympathetic, and sensory - were decreased in old *vs.* young mice. In conjunction, there was a higher incidence of inducible ventricular tachycardia in hearts isolated from old *vs.* young mice.

A time-course experiment revealed that nerve degeneration presented at 16 months prior to the onset of capillary rarefaction at 20 months - suggesting that nerve degeneration is not caused by loss of capillaries but rather may be due to alterations in vascular-derived neuroguidance cues. RNA sequencing (RNA-seq) of cardiac endothelial cells (ECs) isolated from old mice revealed upregulation of genes encoding pathways involved in neuronal death and axon injury, in particular semaphorin 3A (*Sema3a*). Interestingly, prior work had revealed that both deletion and overexpression of Sema3a can cause ventricular arrhythmias and sudden death in mutant mice^[4]^.

To investigate upstream mechanisms of *Sema3a* regulation, Wagner *et al.* identified two micro-RNA (miR)-145 binding sites in the 3′-untranslated region of the *Sema3a* mRNA, the level of which was reduced in old mouse hearts^[3]^. Indeed, *miR-143/145* knockout mice had higher Sema3a levels in a perivascular distribution and reduced axon density. Conversely, endothelial-specific adeno-associated virus (AAV)-mediated *Sema3a* overexpression was sufficient to reduce nerve density *in vivo* in mouse hearts, suggesting that the miR-145/*Sema3a* signaling pathway contributes to loss of nerve density in the aging heart. Analysis of prior singlecell RNA-seq (scRNA-seq) data comparing old *vs*. young mouse hearts^[5]^ revealed that ECs exhibited the greatest upregulation of senescence marker genes. Moreover, the expression of Sema3a and other neurorepelling factors (e.g., Slit3, Netrin-1) was augmented specifically in ECs with the most advanced senescence scores. Taken together, these results indicate that aging results in ECs acquiring a senescent phenotype, which may drive axon degeneration. On the other hand, the mechanisms responsible for miR-145 downregulation in ECs in aging hearts remain unclear.

Lastly, Wagner *et al.* conducted an *in vivo* rescue experiment by administering senolytic drugs Dasatinib and Quercetin, which target apoptotic pathways, to 18-month-old mice for 2 months^[3]^. Senolytic treatment prevented the age-associated reduction in sympathetic and sensory cardiac nerve density in conjunction with attenuation in cardiac EC senescence. At the cellular level, scRNA-seq revealed that Sema3a was repressed in senolytic-treated hearts and that this effect occurred specifically in ECs whereas other cell types implicated in cardiac aging, such as fibroblasts, were unaffected. The senolytic treatment rescued the age-associated decline in heart-rate variability, an indicator of autonomic instability, and decreased ventricular tachycardia inducibility, while improving diastolic function. Interestingly, while the senolytic treatment reduced age-related cardiac fibrosis and macrophage infiltration, neither miR-143/145 deficiency nor premature senescence induced fibrosis or macrophage numbers, suggesting that fibrosis or inflammation are not a major cause of ventricular arrhythmias caused by aging-induced alterations in the miR-145/*Sema3a* axis. Therefore, additional studies will be required to actually elucidate the mechanisms responsible for altered arrhythmogenesis in aging hearts.

Taken together, the study by Wagner *et al.* uncovered a new mechanism that involves aging ECs releasing more Sema3a, which reduces neuronal axon density in the heart, thereby promoting ventricular arrhythmias [Figure 1]^[3]^. These findings are consistent with a prior study showing that cardiomyocyte-specific overexpression of *Sema3a* reduced sympathetic innervation, prolonged action potential duration, catecholamine hypersensitivity, and susceptibility to ventricular tachycardia^[4]^. On the other hand, genetic knockout of *Sema3a* led to sinus bradycardia and early postnatal mortality secondary to stellate ganglia malformation^[4]^. Subsequent studies in a rat model of myocardial infarction reported that *Sema3a* overexpression in the left stellate ganglion reduced sympathetic re-innervation of the infarct margin and reduced susceptibility to ventricular tachycardia^[6]^; the latter finding apparently contradicts the study by Wagner *et al.* While these prior studies established the relevance of semaphorin signaling in relation to cardiac arrhythmogenesis^[3]^, Wagner *et al.* advanced the field by using modern technologies (such as scRNAseq) to demonstrate that senescent ECs are a significant source of semaphorin signaling responsible for changes in innervation^[3]^.

A major appeal of the study by Wagner *et al.* is their finding that aging-related losses in cardiac innervation could be reversed by the administration of senolytic drugs, suggesting that this mechanism is amenable to therapeutic intervention^[3]^. Senolytics are a class of small molecular drugs that selectively clear senescent cells by inducing cell death^[7]^. Indeed, Wagner *et al.* shows that senolytics Dasatinib and Quercetin reduced the number of senescent cells, which was paralleled by an increase in the number of nerve cells^[3]^. Surprisingly, senolytic treatment did not alter capillary density, leading to the question of which cell type(s) were cleared by cell death induced by these drugs. These findings suggest the involvement of another cell type (e.g., pericytes)^[8]^ or an alternative (non-cell death-related) mechanism of action of the senolytics. Thus, while the study by Wagner *et al.* did not establish that the effects of Dasatinib and Quercetin on cardiac function and arrhythmogenesis are a result of eliminating senescent ECs, the normalization of Sema3a, miR-145 levels, and axonal density suggests that normalizing semaphorin signaling in ECs is a significant factor^[3]^.

In conclusion, the study by Wagner *et al.* highlights the need for more aging-related cardiovascular research and the importance of examining the role of various cell types, including neurons and ECs in the heart^[3]^. Additional studies are needed to uncover other EC-dependent mechanisms that underlie cardiac aging aside from the miR-145-*Sema3a* pathway. Additionally, while this study contributes to a growing body of preclinical evidence for the use of senolytics in the aging heart, there has not yet been any clinical evidence that senolytics improve cardiac function in the aging heart.

## Figures and Tables

**Figure 1.**
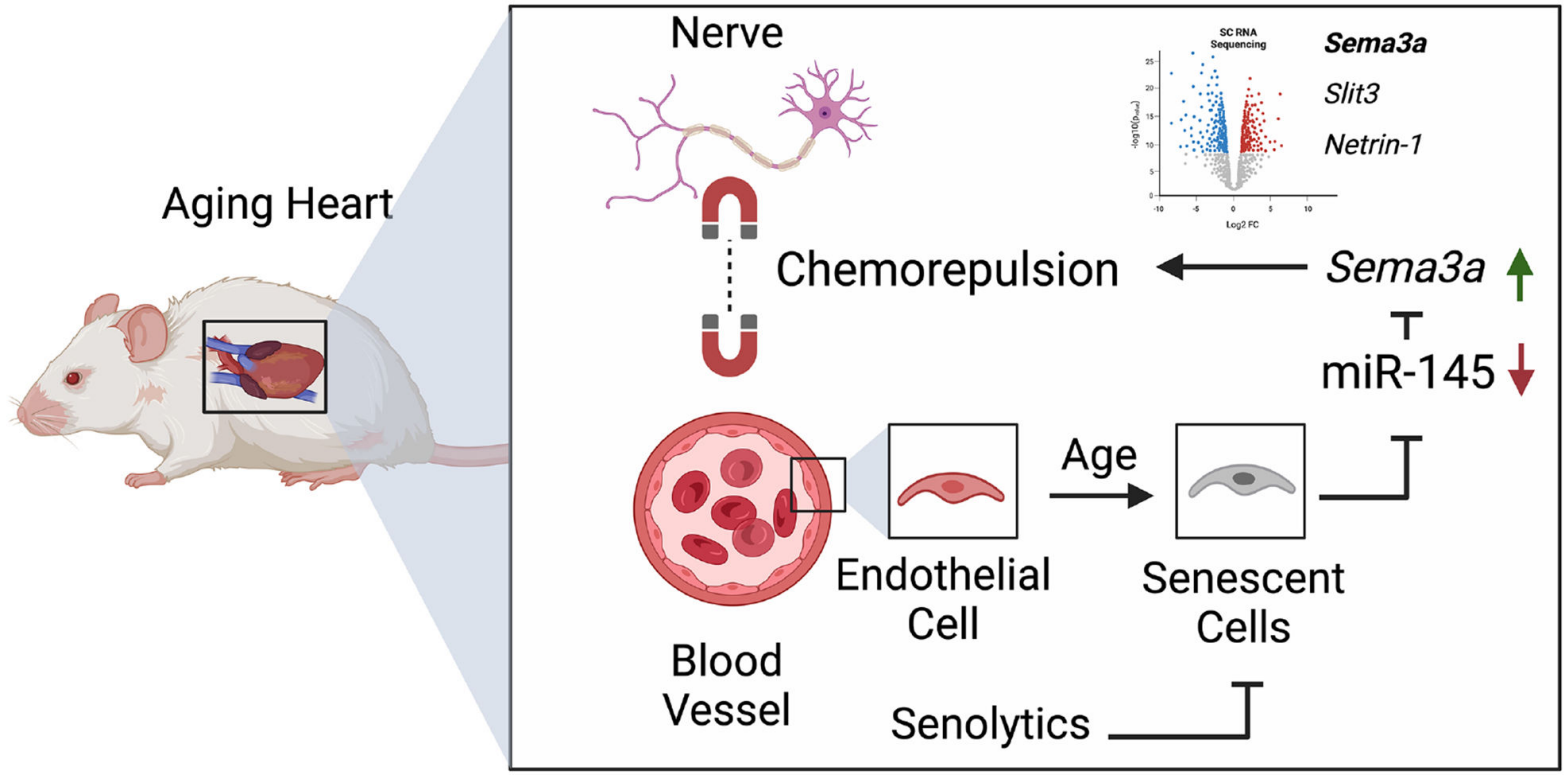
Graphic summary showing that in the aging heart, vascular endothelial cells (ECs) become dysfunctional. The level of microRNA (miR)-145 is reduced in senescent ECs, resulting in increased semaphorin 3A (Sema3a) levels and upregulation of other neurorepelling factors such as Slit3 and Netrin-1. These factors reduce neuronal axon density, promote axon degeneration, and increase the susceptibility to cardiac arrythmias. On the other hand, senolytics can counteract cardiac EC senescence, reduce autonomic instability, and decrease ventricular tachycardia inducibility in mice.
